# The Clinical Utility of Non-invasive Prenatal Testing for Pregnant Women With Different Diagnostic Indications

**DOI:** 10.3389/fgene.2020.00624

**Published:** 2020-06-30

**Authors:** Jianli Zheng, Haiyan Lu, Min Li, Yongjuan Guan, Fangfang Yang, Mengjun Xu, Jingjing Dong, Qinge Zhang, Ning An, Yun Zhou

**Affiliations:** Department of Prenatal Diagnosis, Center of Medical Genetics, Yancheng Maternity and Child Health Care Hospital, Yancheng, China

**Keywords:** non-invasive prenatal testing, trisomy 21, 18, 13, sex chromosome aneuploidies, rare autosomal trisomies, copy number variation

## Abstract

**Background:**

Our aim was to evaluate the clinical utility of non-invasive prenatal testing for pregnant women with different diagnostic indications.

**Methods:**

In eight counties and districts of Yancheng, we studied 13,149 pregnant women with different indications who were offered NIPT for fetal screening, including for sex chromosomal aneuploidies (SCAs), rare autosomal trisomies (RATs), and subchromosomal copy number variations (CNVs). The purpose was to compare the detection of positive predictive values (PPVs) of different indications with the use of NIPT. The results were validated by karyotyping, chromosomal microarray analysis (CMA), or follow-up of pregnancy outcomes.

**Results:**

13,149 maternal plasma samples were sequenced, among which 28 samples (0.2%) failed the sequencing quality control. The remaining 13,121 samples were analyzed, and birth follow-up missed 2,192 samples (16.7%). The PPVs of NIPT results for trisomy 21 (T21) and trisomy 18 (T18) and SCAs were 96.67, 63.64, and 31.34%, respectively. Among the advanced maternal age (AMA), serum screening high risk (SSHR), serum screening intermediate risk (SSIR), and voluntary screening (VS) groups, the PPVs for the common trisomies were 81.25, 85.71, 100, and 70%, respectively; the PPVs for total chromosomal abnormalities were 55.82, 65.22, 23.08, and 36.59%, respectively.

**Conclusion:**

NIPT for T21 and T18 and SCAs screening were ideal, and the PPVs for trisomy 13 (T13), RATs, and CNVs were low. For the AMA and VS groups, NIPT could be used as a first-line screening program; for SSHR and SSIR groups, NIPT could be used as a second-line supplementary screening program.

## Introduction

At present, the total incidence of birth defects in China is about 5.6%, and about 900,000 new birth defects are added every year. Birth defects caused by chromosomal abnormalities are one of the most important types. It is estimated that the incidence rate of trisomy 21 (T21) is approximately one in 800 births (Driscoll and Gross, 2009), but the risk increases with maternal age, reaching one in 35 for a 45-year-old woman (Morris et al., 2002; Savva et al., 2006). The incidence rates of trisomy 18 (T18) and trisomy 13 (T13) are estimated to be approximately one in 6,000 and one in 10,000, respectively (Bianchi et al., 2012). The incidence rate of sex chromosomal aneuploidies (SCAs) is approximately one in 500 births (Nicolaides, 2011). The incidence rate of chromosome microdeletion and microduplication syndrome is about 1/200,000–1/4,000 in births, with the overall incidence rate as high as 1/600 (Wapner et al., 2015). The traditional methods of prenatal diagnosis in China are amniocentesis or umbilical cord blood puncture for pregnant women with high risk, such as advanced maternal age (AMA), high risk of serum screening, and abnormal sonographic indications.

In 2011, detection of maternal plasma cell-free DNA (cfDNA) by massively parallel sequencing came into clinical use in China. NIPT has shown high sensitivity and specificity for the screening of T21, T18, and T13 (Gil et al., 2013; Song et al., 2013; Bianchi et al., 2014; Porreco et al., 2014; Zhang et al., 2015; Manotaya et al., 2016; Fiorentino et al., 2017; Liang et al., 2018). Genome-wide screening studies including for SCAs, rare autosomal trisomies (RATs), and copy number variations (CNVs) have also been carried out in recent years (Fiorentino et al., 2017; Pescia et al., 2017; Liang et al., 2019). Currently, NIPT is offered as an alternative option to serum screening for the prenatal detection of aneuploidies in some hospitals, but it is a complex and relatively expensive testing technology. The expense means it is not always taken up. Furthermore, prenatal screening for chromosome abnormalities remains non-compulsory and is not covered by the healthcare system in China.

Previous NIPT studies have classified the pregnant population into two groups: high risk and low risk, maternal age ≥ 35 years and < 35 years (Song et al., 2013; Bianchi et al., 2014). Although previous study populations were classified according to the prenatal diagnostic indications (Song et al., 2013; Porreco et al., 2014), the positive predictive value (PPV) and negative predictive value (NPV) of different indications were not analyzed.

In our study, the NIPT results and pregnancy outcomes with different indications were calculated to guide clinicians to design appropriate screening and diagnostic programs for pregnant women so as to effectively reduce the incidence of birth defects.

## Materials and Methods

### Study Population

From January 2015 through December 2017, we implemented NIPT screening for pregnant women in eight counties and districts of Yancheng. NIPT was offered to 13,149 pregnant women with different indications in the Center of Prenatal Diagnosis of Yancheng Maternity and Child Health Care Hospital. According to current standard practice in China, all participants were at least 12 weeks pregnant. They were divided according to prenatal diagnostic indications: AMA (≥35), serum screening high risk (SSHR, cutoff ≥ 1/270), serum screening intermediate risk (SSIR, cutoff 1/1,000–1/270), voluntary screening (VS), abnormal soft indexes of ultrasound, twin pregnancy, adverse pregnancy, and childbirth history. All the pregnant women gave informed consent before blood collection. The clinical study was approved by the Medical Ethics Committee of the hospital.

### Maternal Plasma DNA Processing and Sequencing

10 ml peripheral blood from each participant was drawn into an EDTA-containing vacutainer tube. Within 8 h of collection, the maternal blood samples were centrifuged and extracted. The plasma was frozen and shipped to Berry Genomics in Beijing for DNA extraction and subsequent sequencing analysis.

Cell-free DNA was purified from the plasma fraction using the fetal chromosome aneuploidy test kit developed by Berry Genomics (Beijing, China). Approximately 10 ng of cfDNA was then used to construct cfDNA libraries. After quantification, libraries were tag sequenced on the NextSeq CN500 platform (Illumina) to generate approximately 5M reads per sample.

The data meeting quality control standards was analyzed. Calculating a Z score per chromosome, samples with a normalized chromosome value of 3.0 or less were classified as unaffected.

### Clinical Outcomes

Analysis was performed for all samples on chromosomes 21, 18, 13, SCAs, RATs, and CNVs. Pregnant women with positive NIPT results were advised to undergo amniocentesis. Prenatal diagnosis methods included karyotyping and/or chromosomal microarray analysis (CMA).

Amniotic fluid cell culture was performed according to the standard techniques. Routine G-bands by trypsin using Giemsa (GTG) analysis at 400-band resolution was used to prepare the amniotic cell chromosome specimens (Zheng et al., 2019).

Human cyto12 SNP array (Illumina, United States) comprising around 300,000 SNP probes was applied for whole-genome scan on the amniotic cell DNA of the fetus. SNP-array tests were performed according to the manufacturer’s protocol (Illumina, United States); molecular karyotype analysis was carried out by KaryoStudio V 1.4.3.0 (Illumina, United States). Databases such as DECIPHER^1^, DGV^2^, UCSC^3^, and OMIM^4^ were used as references to evaluate the array data and analyze genotype–phenotype correlations (Zheng et al., 2019).

Three months after the diagnosis, these pregnant women were followed up for pregnancy outcomes. Pregnant women with negative NIPT results were recommended to continue routine prenatal examinations. These cases were interviewed by telephone 3 months after delivery to obtain information on neonatal outcome.

### Statistical Analysis

The Clopper–Pearson method was used to calculate the performance of the test (sensitivity, specificity, PPV, and NPV) and exact 95% confidence intervals. Comparisons between groups were performed using chi-square test or Fisher’s exact test and a *P*-value of ≤ 0.05 was defined as statistically significant.

## Results

A total of 13,149 maternal plasma samples were sequenced, among which 28 samples (0.2%) failed the sequencing quality control due to inadequate fetal fraction (lower than 4%). The remaining 13,121 samples were analyzed. The average age of the analyzed participants was 28 years (range 17–48 years), and mean gestational age was 17^+2^ weeks (range 12–29 weeks).

**FIGURE 1:**
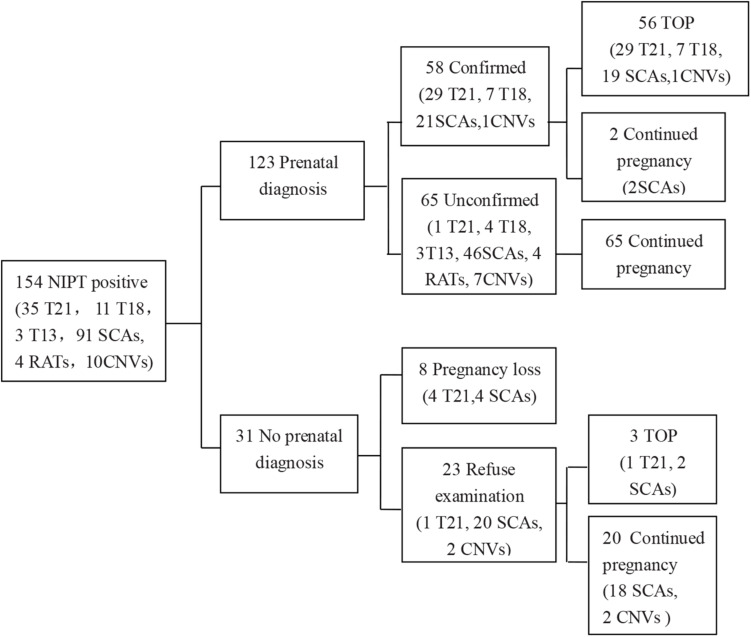
Detailed outcome results of NIPT-positive cases. T21, trisomy 21; T18, trisomy 18; T13, trisomy 13; SCA, sex chromosomal aneuploidy; CNV, copy number variation; TOP, termination of pregnancy. Information in the brackets indicated the case numbers with different NIPT results.

Of the 13,121 participants, clinically relevant chromosomal abnormalities were detected in 154 (1.17%) pregnancies. Among them, 140 cases were common aneuploidies, four cases were RATs, and 10 cases were CNVs. Among all the NIPT-positive cases, amniocentesis was performed in 123 cases and 58 cases were confirmed by karyotyping or CMA (Table 1), 57 cases of which were common aneuploidies, and one case was of segmental imbalances. In total, the acceptance rate of amniocentesis was 79.87% (123/154) and the PPV of chromosomal abnormalities was 47.15% (58/123). 56 (96.55%) of the confirmed cases chose termination of pregnancy (TOP), while two fetuses with karyotype 47,XXX and 45,X/46,XX were born (Figure 1).

**TABLE 1:** Demographic characteristics of pregnant women undergoing NIPT and confirmatory results from January 2015 through December 2017.

Indications	Proportion	NIPT positive	NIPT positive	T21	T18	T13	SCA	RAT	CNV
	(%)	Rate (%)	TP/IPD	TP/IPD	TP/IPD	TP/IPD	TP/IPD	TP/IPD	TP/IPD
Total	13,121	1.17	154 58/123	35 29/30	11 7/11	3 0/3	91 21/67	4 0/4	10 1/8
Advanced maternal age	3,079(23.47)	1.56	48 24/43	14 12/13	2 1/2	1 0/1	25 10/22	1 0/1	5 1/4
Serum screening high risk	1,877(14.31)	1.28	24 15/23	9 9/9	4 3/4	1 0/1	9 3/9	0 NA	1 NA
Serum screening intermediate risk	2,710(20.65)	0.63	17 3/13	3 3/3	0 NA	0 NA	14 0/10	0 NA	0 NA
Voluntary screening	4,675(35.63)	1.18	55 15/41	5 4/4	5 3/5	1 0/1	38 8/25	3 0/3	3 0/3
Abnormal ultrasound soft indexes	295(2.25)	1.69	5 0/2	2 NA	0 NA	0 NA	2 0/1	0 NA	1 0/1
Twin pregnancy	114(0.87)	3.51	4 1/1	2 1/1	0 NA	0 NA	2 NA	0 NA	0 NA
Adverse pregnancy history	371(2.83)	0.27	1 NA	0 NA	0 NA	0 NA	1 NA	0 NA	0 NA

*Abbreviations: TP, true positive; IPD, invasive prenatal diagnosis; T13, trisomy 13; T18, trisomy 18; T21, trisomy 21; SCA, sex chromosomal aneuploidy; RAT, rare aneuploidy trisomy; CNV, copy number variation; NA, not applicable.*

### CfDNA Screening Results With Different Indications

A total of 13,121 pregnant women were offered NIPT. The NIPT results of each indication group are shown in Table 1. Among the 3,079 pregnant women of the AMA group, 12 T21, one T18, 10 SCAs, and one CNV were found by NIPT screening and prenatal diagnosis of amniotic fluid. Of the 1,877 pregnancies with SSHR, nine T21, three T18, and three SCAs were found. In the 2,710 pregnant women with SSIR, three T21 were diagnosed. In the 4,675 pregnant women of voluntary NIPT screening group, four T21, three T18, and eight SCAs were diagnosed. In addition, one of 114 twin pregnancies was diagnosed as T21. In the abnormal ultrasound soft indexes group and the abnormal history of pregnancy group, no chromosomal abnormalities were diagnosed.

In the seven indications, the number of VS, AMA, and SSIR groups ranked in the top three, accounting for 35.63% (4,675/13,121), 23.47% (3,079/13,121), and 20.65% (2,710/13,121), respectively (Table 1). Detection rates of chromosomal abnormalities in different subgroups are listed in Table 2. Pregnancies in twin pregnancy group (0.88%), SSHR group (0.80%), and AMA group (0.78%) were discovered to have high detection rates of chromosomal abnormalities. Compared with the VS group, there were statistically significant differences in the detection rates of chromosomal abnormalities for SSHR group (0.80% vs 0.32%, *P* < 0.05) and AMA group (0.78% vs 0.32%, *P* < 0.05). Among the AMA, SSHR, SSIR, and VS groups, the PPVs for the common trisomies were 81.25, 85.71, 100, and 70%, respectively; the total PPVs for all chromosomal abnormalities were 55.82, 65.22, 23.08, and 36.59%, respectively.

**TABLE 2:** Detection rates of chromosomal abnormalities in different subgroups.

Groups of NIPT	*n*	Chromosomal abnormalities (%)	*P*-value (vs voluntary screening)
Advanced maternal age	3,079	24 (0.78)	<0.05
Serum screening high risk	1,877	15 (0.80)	<0.05
Serum screening intermediate risk	2,710	3 (0.11)	0.128
Voluntary screening	4,675	15 (0.32)	
Abnormal ultrasound soft indexes	295	0	/
Twin pregnancy	114	1 (0.88)	0.845
Adverse pregnancy history	371	0	/
Total	13,121	58 (0.44)	

*The detection rate of chromosomal abnormalities in the voluntary screening group is close to that of the whole population.*

### T21, T18, T13, and SCA Detection

Among the 13,121 reportable samples of NIPT, 35 cases were classified with T21, 11 with T18, three with T13, and 91 with SCAs. The pregnant women who underwent amniocentesis amounted to 30 with T21, 11 with T18, three with T13, and 67 with SCAs. 29 T21, 7 T18, and 21 SCAs were confirmed as true positives. The acceptance rate of amniocentesis for common trisomies was 89.80% (44/49) and 73.63% (67/91) for SCAs. The PPV for common trisomies was 81.82% (36/44) and 31.34% (21/67) for SCAs.

Of the 13,121 women with NIPT results, 10,929 (83.3%) cases with low-risk results were successfully followed-up through telephone survey (Figure 1). From follow-up tests, Table 2 summarizes the performance of NIPT for common aneuploidies. For T21, T18, T13, and SCAs, the sensitivity was 100%, 100%, NA, and 100%; the specificity was 99.99, 99.96, 99.97, and 99.58%; and the PPV was 96.67, 63.64, 0, and 31.34%, respectively. One case of SCAs was confirmed by CMA and FISH as karyotype 45, X [5]/46, X, del (Y) (q11.2) [11]/46, X, and idic (Y) (q11.2) [14]; another case was confirmed by CMA as karyotype 45, X [4]/46, XX [36]. Table 3 shows karyotype and CMA verification of eight NIPT-positive results.

**TABLE 3:** Karyotype and CMA verification of eight NIPT positive results.

Age	Week	NIPT result	Karyotyre	CMA result
1.33	16^+3^	45,X	45,X[30]/46,XY, del(Y)(q11)[10]	46,XY, del(Y)(q11.22-q11.23,9.0M)
2.24	17^+6^	45,X	45,X[5]/46,X, +marl [11]/46, X, +mar2[14]	47,XYY, del(Y)(q11.22-q12,7.8M)
3.26	18^+1^	45,X	45, X [4]/46, XX [36]	45, X (20%)/46, XX (80%)
4.38	21^+5^	45,X	46,XX	46,XX, dup(8)(p23.2,2.2M), dup(18)(q22.1,1.4M)
5.28	16	45,X	46,XX	46,XX
6.40	18^+2^	del4 and dup1	46,XX	46,XX
7.31	17^+3^	del14 and 45,X	46,XY	46,XY
8.30	20^+5^	del13, del14, and 47,XXY	46,XY	46,XY

### CfDNA Screening Results of RATs and CNVs

Among the 13,121 reportable samples following cfDNA analysis, RATs was identified in four samples and no case was confirmed. Segmental chromosomal imbalances were found in 10 samples, and eight cases underwent amniocentesis. Only one case of CNVs was confirmed by invasive prenatal diagnosis (IPD). In addition, one case showed chromosome 3 and 5 abnormalities; the fetus was confirmed normal, but the mother was confirmed with tumors. For RATs and CNVs, the sensitivity was NA and 100%, the specificity was 99.96 and 99.94%, and the PPV was 0 and 12.50%, respectively (Table 4). We assumed that all imbalances had been identified in the 3 months after birth.

**TABLE 4:** Performance of NIPT in detecting trisomies 21, 18, 13, SCAs, RATs, and CNVs.

	TP	FP/FPR	Sensitivity (95% CI)	PPV (95% CI)	TN	FN/FNR	Specificity (95% CI)	NPV (95% CI)	Incidence
T21	29	1/0.01%	100%	96.67%	10924	0/0%	99.99%	100%	0.22%
			(85.44–100%)	(80.95–99.83%)			(99.94–100%)	(99.96–100%)	
T18	7	4/0.04%	100%	63.64%	10929	0/0%	99.96%	100%	0.05%
			(56.09–100%)	(31.61–87.63%)			(99.90–99.99%)	(99.96–100%)	
T13	0	3/0.03%	NA	0%	10929	0/100%	99.97%	100%	0%
				(0–69.00%)			(99.91–99.99%)	(99.96–100%)	
SCAs	21	46/0.42%	100%	31.34%	10905	0/0%	99.58%	100%	0.16%
			(80.76–100%)	(20.87–43.97%)			(99.44–99.69%)	(99.96–100%)	
RATs	0	4/0.04%	NA	0%	10929	0/100%	99.96%	100%	0%
				(0–60.42%)			(99.90–99.99%)	(99.96–100%)	
CNVs	1	7/0.06%	100%	12.50%	10927	0/0%	99.94%	100%	0.01%
			(5.46–100%)	(0.66–53.32%)			(99.86–99.97%)	(99.96–100%)	

*Abbreviations: CI, confidence interval; TP, true positive; FP, false positive; FPR, false positive rate; PPV, positive predictive value; TN, true negative; FN, false negative; FNR, false negative rate; NPV, negative predictive value; NA, not applicable.*

### Follow-Up Outcomes

**FIGURE 2:**
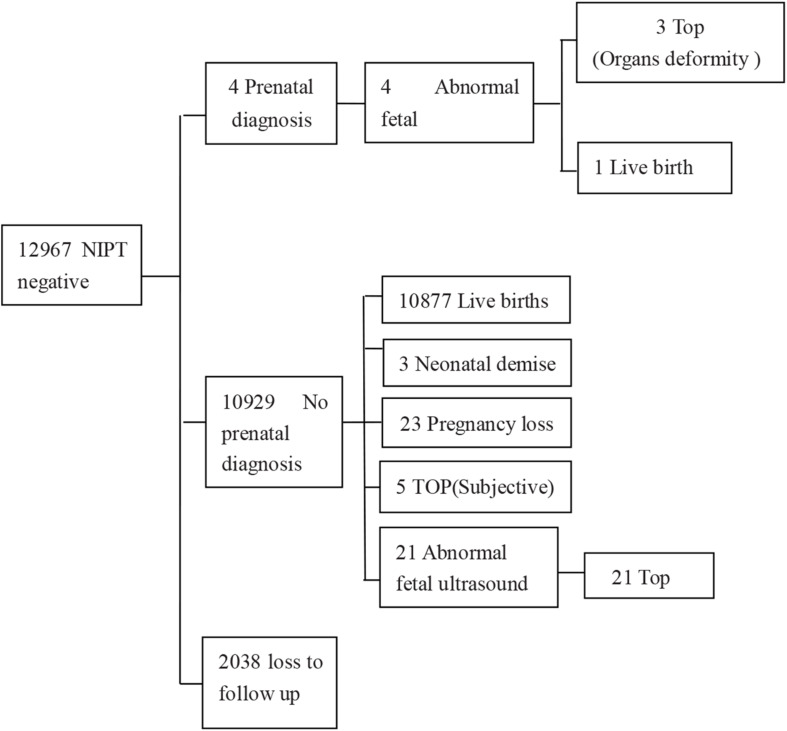
Detailed outcome of NIPT negative results. TOP, termination of pregnancy.

The 13,121 analyzed cases were interviewed by telephone, and birth follow-up missed 2,192 samples (16.7%) (Figure 2). Our cohort also included 28 cases who encountered a test failure and the follow-up results revealed that 26 of them had live births with normal neonatal physical examinations. One woman underwent TOP because of an abnormal fetal ultrasound result and one missed follow-up.

## Discussion

### Principal Findings of the Study

NIPT has been widely used to screen for T21, T18, and T13 in the past few years (Gil et al., 2013; Song et al., 2013; Bianchi et al., 2014; Wapner et al., 2015; Zhang et al., 2015; Manotaya et al., 2016; Liang et al., 2018), yet clinical studies about cfDNA screening and its efficacy in pregnant women with different indications are lacking. There are clear criteria for the clinical application of NIPT in China, which are managed according to the applicable population, cautious population, and unsuitable population. The applicable population includes three categories: (1) SSIR (cutoff 1/1,000–1/270); (2) pregnant women with contraindications of interventional prenatal diagnosis (such as threatened abortion, fever, bleeding tendency, etc.); and (3) pregnant women missed serum screening. The cautious population includes seven categories: (1) pregnant women with prenatal screening high risk in early and middle pregnancy; (2) the expected age of delivery ≥ 35 years; (3) severe obesity (BMI > 40); (4) conceived by *in vitro* fertilization and embryo transfer; (5) there is a history of fetal delivery with chromosomal abnormalities; (6) twin and multiple pregnancies; and (7) other circumstances that may affect the accuracy of the results in the opinion of the doctor. The unsuitable population includes seven categories: (1) gestational age < 12 weeks; (2) the couple had definite chromosomal abnormalities; (3) within 1 year, pregnant women received allogeneic blood transfusion, transplantation, allogeneic cell therapy, etc.; (4) fetal ultrasound examination indicated structural abnormalities; (5) having a family history of genetic disease or suggesting a high risk of genetic disease in the fetus; (6) pregnancy with malignant tumor; and (7) other circumstances that the doctor thinks have a significant impact on the accuracy of the results.

This study focused on a comparative analysis of different application indications and NIPT test results to explore the application value of cfDNA testing for different screening indications, which could provide a reference for further standardization and rational use of this technology.

With the two-child policy, maternal and infant safety has attracted more attention, especially birth defects. According to Chinese maternal and infant healthcare law, IPD should be carried out for pregnant women of AMA. However, in clinical practice, although fully informed, only some of these pregnant women are really willing to accept IPD. Baker et al. (2004) found that 47% of older pregnant women would accept amniocentesis directly, which resulted in the majority of the older pregnant women not receiving effective follow-up treatment. In clinical practice, many older pregnant women are willing to accept NIPT as a non-invasive and accurate screening. In our study, the proportion of AMA indications in the NIPT screening population was the second highest, reaching 23.47%. NIPT found 17 cases with high risk of the three common trisomies. 16 cases underwent amniocentesis, and 13 cases were diagnosed. One case had labor induced directly. The PPV for the three common trisomies was 81.25% (13/16). Additionally, NIPT found 25 cases of SCAs, 22 cases underwent IPD, and 10 cases were confirmed. The PPV for SCAs was 45.45% (10/22). Among one RATs and five CNVs cases, only one case of CNVs was confirmed in five amniocenteses. The PPV for RATs and CNVs was 20% (1/5). Of all the confirmed T21 cases, 41.38% (12/29) was from the AMA group, and no missed diagnosis has been found in the follow-up so far. The study shows that pregnant women in AMA group have high acceptance of NIPT, and the screening effect is obvious. So NIPT can be used as an effective screening method for this population, thus reducing the occurrence of missed diagnosis of T21, T18, and T13.

There were 1,877 pregnant women with SSHR in this study. NIPT indicated 14 cases with high risk of T21, T18, and T13; amniocentesis was performed in all 14 cases, and 13 cases were confirmed. 31.03% (9/29) of the diagnosed T21 and 42.86% (3/7) of diagnosed T18 came from this group. The PPV was 85.71% (12/14) for T21, T18, and T13. NIPT found nine cases of SCAs; all cases underwent IPD and confirmed three cases. The PPV for SCAs was 33.33% (3/9). Clinical practice has found that 64% of pregnant women with SSHR accept amniocentesis; another 36% fear that amniotic fluid puncture may cause miscarriage or harm the fetus (Baker et al., 2004; Grinshpun-Cohen et al., 2015). The study reveals that NIPT has high accuracy, and it can be used as a second-line non-invasive screening method before amniocentesis for the serum high-risk population.

In 2016, China promulgated NIPT regulations which included the intermediate-risk population. In conventional maternal serum screening, the high-risk group usually accounts for 3–5%, and the intermediate-risk group accounts for 8–9% (Chitty et al., 2016). Therefore, the population of the intermediate-risk group is at least 1.5 times higher than that of the high-risk group. In our study, there were 2,710 cases in the intermediate-risk group, which is exactly 1.44 times that of the high-risk group. NIPT found three cases with high risk of T21, of which all were diagnosed. The PPV was 100% for T21. NIPT revealed 14 cases of SCAs; 10 cases underwent amniocentesis, and none were confirmed. In this study, 10.34% (3/29) of confirmed T21 cases were from the SSIR group. If these three pregnant women had not undergone NIPT, all of them would have missed diagnosis. The incidence of abnormalities for the group was 0.11% (3/2710). Compared to the VS group, the detection rate was not statistically significant (0.11 vs 0.32%, *P* = 0.128). However, the study means that if the intermediate-risk population does not undergo effective testing may miss diagnosis. Therefore, we should strengthen genetic counseling and follow-up management of the intermediate-risk population, which would be one of the most effective measures to further reduce birth defects.

The voluntary NIPT screening group comprised 4,675 cases, accounting for the highest proportion of the participants, reaching 35.63%. There were three types of pregnant women: with no serum screening, low risk of serum screening, and missed the serum screening. The high percentage reflected their high acceptance of NIPT. In a sense, NIPT serves as a first-line screening method and a remedial screening method for the voluntary group. NIPT found 11 cases with high risk of T21, T18, and T13. Except for one high-risk pregnant woman with T21 who had spontaneous abortion, the remaining 10 cases were diagnosed as T21 in four cases and T18 in three cases by amniocentesis. The PPV for the common trisomies was 70% in this group. NIPT showed 38 cases with SCAs, 25 cases underwent amniocentesis, and eight cases were confirmed. The PPV for SCAs was 32% (8/25). Three cases of RATs and three cases of CNVs high-risk were not diagnosed. This suggests that if the 15 confirmed cases in the group were not screening using NIPT, follow-up ultrasound was not done or did not indicate abnormalities, and missed diagnosis may occur. Again, this reminds us to strengthen the management of ultrasonography in mid and late pregnancy to further reduce birth defects.

In the abnormal ultrasound soft index group, NIPT showed two cases with T21, two cases with SCAs, and one case with CNVs. No case was diagnosed. In the twin pregnancy group, NIPT showed two cases with T21 and two cases with SCAs. Only one case with T21 underwent amniocentesis and was diagnosed, and subsequently abortion was induced. In the group of adverse pregnancy and childbirth history, NIPT indicated one case with SCAs, but no further examination was performed. There were 780 pregnant women in these three groups, accounting for only 5.94% of the participants. More data are needed to predict the efficacy of NIPT on these indications for the time being.

In this study, we summarized the clinical data of 13,121 cfDNA screening cases in our center. Our results revealed high sensitivity and specificity for T21, T18, SCAs, and CNVs, which is in line with previous studies (Porreco et al., 2014; Manotaya et al., 2016; Fiorentino et al., 2017; Liang et al., 2018). As for T13 and RATs, no positive results were found, which could be due to the low number of T13 and RATs cases detected in the participants. Similarly, for the PPVs, our results revealed a comparable number for T21, T18, and SCAs with other studies, but the PPV for T13, CNVs, and RATs was much lower in our population compared with others (Zhang et al., 2015; Petersen et al., 2017; Liang et al., 2019). The reasons are as follows:(1) it may be due to maternal chromosome abnormality, confined placental mosaicism (CPM) or fetal mosaicism (Bianchi et al., 2012; Faas et al., 2012; Hall et al., 2013); (2) it may be due to the low resolution of karyotype diagnosis, which requires the use of higher resolution diagnostic methods such as CMA (Srinivasan et al., 2013); (3) the sequencing depth in our study was only 5M reads per sample, lower than the 20–30M reads per sample in some studies (Petersen et al., 2017; Liang et al., 2019). Additionally, NIPT found a pregnant woman with chromosome 3 and 5 abnormalities, and subsequently she was diagnosed with malignancy. Osborne et al. (2013) and Bianchi et al. (2015) found similar cases in previous studies.

## Conclusion

In summary, NIPT screening for T21, T18, and SCAs in our study was ideal. The study also suggests that NIPT screening is clinically effective for the common trisomies in pregnant women with different indications. Although the PPVs for T13, RATs, and CNVs were low, it provides a feasible screening method, and we will further optimize this method to improve the detection accuracy. Furthermore, it suggests that our classified prenatal diagnostic indications have important implications in preventing birth defects.

## Data Availability Statement

The datasets for this article are not publicly available because of privacy concerns. Requests to access the datasets should be directed to JZ, zjl-0221@163.com.

## Ethics Statement

The studies involving human participants were reviewed and approved by the Medical Ethics Committee of Yancheng Maternity and Child Health Care Hospital.

## Author Contributions

All the authors listed approved the manuscript.

## Conflict of Interest

The authors declare that the research was conducted in the absence of any commercial or financial relationships that could be construed as a potential conflict of interest.

## Footnotes

1 http://decipher.sanger.ac.uk/

2 http://projects.tcag.ca/variation

3 http://genome.ucsc.edu/

4 http://www.ncbi.nlm.nih.gov/omim
